# Supplementary material for: Rab1b and ARF5 are novel RNA-binding proteins involved in FMDV IRES–driven RNA localization
Source: Life Sci Alliance. 2019 Jan 17;2(1):e201800131. doi: 10.26508/lsa.201800131 (PMC6337736; doi:10.26508/lsa.201800131)
Supplement: Supplementary file 2 [file LSA-2018-00131_Table_S1.doc]

**Table S1. Oligonucleotides**

| **Plasmid** | **PCR template** | **Oligonucleotide** | **Sequence (5´-3´)** |
| --- | --- | --- | --- |
| pBSMrnaStrep/D3 | pBIC | tRNA-1s | GGGGTCGACGTGTTTGGCTCCACGCTCG |
|  |  | tRNA-2as | GACATTGAAACTGGTACCCACACACGACGTCCCG |
| pBSMrnaStrep/SL123 | pBIC | tRNA-3s | GGGGTCGACTGCTTCGTAGCGGAGCATGACGG |
|  |  | tRNA-4as | GCAACCCCAGCACGGCGGACGTCCCG |
| pBSMrnaStrep/SL3abc | pBIC | tRNA-5s | CACTGTCGACTCGTAG |
|  |  | tRNA-6as | AGACGTCGTGCTG |
| pBSMrnaStrep/SL3a |  | tRNA-7s | TCGAGTGGGAACTCCTCCTTGGTAACAAGGACCCACGGGACGT |
|  |  | tRNA-8as | CCCGTGGGTCCTTGTTACCAAGGAGGAGTTCCCAC |
| pTaggedCAP | pTagged-FMDV | Mut-1s | CCTTTACAATTAATGACCCTGAATTCATGGAAGACGCCAAAAAC |
|  |  | Mut-2as | ATGTTTTTGGCGTCTTCCATGAATTCAGGGTCATTAATTGTAAA |
| peGFP-N1-Rab1b | pPB-N-His-Rab1b | C1-GFPRab-s | CTCGAGCTATGAACCCCGAATATGACTACC |
|  |  | C1-GFPRab-as | GGATCCCTAGCAACAGCCACCG |
| peGFP-N1-Rab1bDN | peGFP-N1-Rab1b | Rab1bS22Ns | CCGCAGGAGCAGGCAGTTCTTGCCCACGCCTGA |
|  |  | Rab1bS22Nas | TCAGGCGTGGGCAAGAACTGCCTGCTCCTGCGG |
